# Rendering hydrophobic nanoclusters water-soluble and biocompatible†

**DOI:** 10.1039/d0sc01055c

**Published:** 2020-04-22

**Authors:** Xi Kang, Xiao Wei, Pan Xiang, Xiaohe Tian, Zewen Zuo, Fengqi Song, Shuxin Wang, Manzhou Zhu

**Affiliations:** Department of Chemistry and Centre for Atomic Engineering of Advanced Materials, Anhui Province Key Laboratory of Chemistry for Inorganic/Organic Hybrid Functionalized Materials, Key Laboratory of Structure and Functional Regulation of Hybrid Materials of Ministry of Education, Anhui University Hefei 230601 P. R. China ixing@ahu.edu.cn zmz@ahu.edu.cn; School of Life Sciences, Anhui University Hefei 230601 P. R. China; National Laboratory of Solid State Microstructures, Collaborative Innovation Center of Advanced Microstructures, School of Physics, Nanjing University Nanjing 210093 P. R. China; Atomic Manufacture Institute Nanjing 211805 P. R. China

## Abstract

Hydrophobic and hydrophilic nanoclusters embody complementary superiorities. The means to amalgamate these superiorities, *i.e.*, the atomic precision of hydrophobic clusters and the water dissolvability of hydrophilic clusters, remains challenging. This work presents a versatile strategy to render hydrophobic nanoclusters water-soluble—the micellization of nanoclusters in the presence of solvent-conjoined Na^+^ cations—which overcomes the above major challenge. Specifically, although [Ag_29_(SSR)_12_(PPh_3_)_4_]^3−^ nanoclusters are absolutely hydrophobic, they show good dissolvability in aqueous solution in the presence of solvent-conjoined Na^+^ cations (Na_1_(NMP)_5_ or Na_3_(DMF)_12_). Such cations act as both counterions of these nanoclusters and surface cosolvent of cluster-based micelles in the aqueous phase. A combination of DLS (dynamic light scattering) and aberration-corrected HAADF-STEM (high angle annular dark field detector scanning transmission electron microscopy) measurements unambiguously shows that the phase-transfer of hydrophobic Ag_29_ into water is triggered by the micellization of nanoclusters. Owing to the excellent water solubility and stability of [Ag_29_(SSR)_12_(PPh_3_)_4_]^3−^[Na_1_(NMP)_5_]_3_^+^ in H_2_O, its performance in cell staining has been evaluated. Furthermore, the general applicability of the micellization strategy has been verified. Overall, this work presents a convenient and efficient approach for the preparation of cluster-based, biocompatible nanomaterials.

## Abstract 2

The presence of solvent-conjoined cations, [Na_1_(NMP)_5_]^+^ or [Na_3_(DMF)_12_]^3+^, induces the micellization of hydrophobic nanoclusters, rendering these nanoclusters water-soluble and biocompatible.

## Graphical abstract

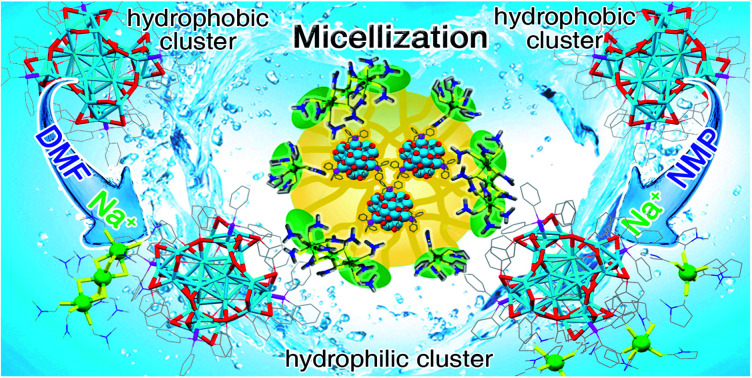

## 1 Introduction

Metal nanoclusters, benefiting from their atomically precise structures, fascinating properties, and potential applications, are an emerging class of modular nanomaterials.^1–11^ Recent years have witnessed significant research efforts on the controllable synthesis and the structural determination of metal nanoclusters.^12–34^ Typically, the great nanocluster family encompasses both hydrophobic and hydrophilic clusters.

(i) Owing to their precise structures, hydrophobic nanoclusters are mostly researched for fathoming structural evolutions and structure–property correlations at the atomic level.^1^ Based on such knowledge and understanding of mechanisms underlying cluster performances, several efficient approaches have been put forward to accurately dictate the chemical–physical performances of metal nanoclusters.^1–11,35–42^ However, in consideration of their hydrophobicity, these nanoclusters are less likely directly applicable to water-phase applications, such as chemical sensing, bio-imaging, bio-labeling, and biotherapy, to name a few.

(ii) On the other hand, although several hydrophilic nanoclusters are not that precise in terms of structures and compositions (even some of them are poly-dispersed), they are much more suitable for the aforementioned bio-applications owing to their water dissolvability.^43–51^ In addition, hydrophilic nanoclusters benefit from ultra-small sizes, non-toxicity, strong photo-stability, good biocompatibility, and potential anti-cancer activity, and their use in biological applications is thus highly promising.^43–51^ However, drawbacks of known hydrophilic nanoclusters also exist—their properties are even harder to precisely control than in hydrophobic nanoclusters, and their imprecise structures preclude quantitative tracking in cells or organisms. These drawbacks greatly impede the practical uses of hydrophilic nanoclusters in bio-applications.

Indeed, hydrophobic and hydrophilic nanoclusters embody complementary superiorities. Accordingly, the means to transfer atomically precise, hydrophobic nanoclusters into water, and then exploit them for aqueous-phase applications, is anticipated to overcome the aforementioned drawbacks, and should be an important goal in nanocluster science. Herein, we report a versatile strategy to render hydrophobic nanoclusters water-soluble—the micellization of these nanoclusters in the presence of solvent-conjoined Na^+^ cations. Specifically, the [Ag_29_(SSR)_12_(PPh_3_)_4_]^3−^ (SSR = 1,3-benzene dithiol) nanocluster is absolutely hydrophobic; however, in the presence of solvent-conjoined cations (Na_1_(NMP)_5_ or Na_3_(DMF)_12_) as counterions, Ag_29_@Na compounds ([Ag_29_(SSR)_12_(PPh_3_)_4_]^3−^[Na_1_(NMP)_5_]^+^_3_ or [Ag_29_(SSR)_12_(PPh_3_)_4_]^3−^[Na_3_(DMF)_12_]^3+^, **Ag29-Na1** or **Ag29-Na3** hereafter because they contain three Na^+^ monomers or one [Na_3_]^3+^ trimer, respectively) show good dissolvability in aqueous solution. A combination of DLS (dynamic light scattering) and aberration-corrected HAADF-STEM (high angle annular dark field detector scanning transmission electron microscopy) measurements unambiguously shows that the phase-transfer of hydrophobic Ag_29_ into water is triggered by the micellization of nanoclusters. Owing to the excellent water dissolvability and stability of **Ag29-Na1**, its performance in cell staining is evaluated, and such cluster-based micelles show specific selectivity in staining lysosomes. Furthermore, the general applicability of this micellization method in the nanocluster field has been verified, based on several other negative-charged nanoclusters, which further demonstrates the significance of this method in the preparation of cluster-based, biocompatible nanomaterials.

## 2 Experimental methods

### Materials

All reagents were purchased from Sigma-Aldrich and used without further purification: silver nitrate (AgNO_3_, 99%, metal basis), triphenylphosphine (PPh_3_, 99%), 1,3-benzene dithiol (SSR, 99%), sodium borohydride (NaBH_4_, 99.9%), sodium acetate (CH_3_COONa, 99%), methylene chloride (CH_2_Cl_2_, HPLC, Aldrich), methanol (CH_3_OH, HPLC, Aldrich), *N*,*N*-dimethylformamide (DMF, HPLC, Aldrich), *N*-methyl-2-pyrrolidone (NMP, HPLC, Aldrich), and ethyl ether ((C_2_H_5_)_2_O, HPLC, Aldrich).

### Synthesis of [Ag_29_(SSR)_12_(PPh_3_)_4_]^3−^

The preparation of [Ag_29_(SSR)_12_(PPh_3_)_4_]^3−^ was based on the reported method of the Bakr and the Pradeep groups.^52,53^

### Synthesis of [Ag_29_(SSR)_12_(PPh_3_)_4_]^3−^[Na_1_(NMP)_5_]^+^_3_ (*i.e.*, **Ag29-Na1**)

20 mg of [Ag_29_(SSR)_12_(PPh_3_)_4_]^3−^ was dissolved in 5 mL of NMP, and 2 mg of CH_3_COONa was added under vigorous stirring at 273 K (ice-bath). After 30 min, the organic layer was separated, which produced the **Ag29-Na1** nanocluster. The yield was 95% based on the Ag element (calculated from the Ag_29_(SSR)_12_(PPh_3_)_4_). This NMP solution of **Ag29-Na1** was directly used for the crystallization and characterization.

### Synthesis of [Ag_29_(SSR)_12_(PPh_3_)_4_]^3−^[Na_3_(DMF)_12_]^3+^ (*i.e.*, **Ag29-Na3**)

50 mg of the **Ag29-Na1** crystal was dissolved in 5 mL of DMF under vigorous stirring at 273 K (ice-bath). This DMF solution was poured into 200 mL of CH_2_Cl_2_, and the precipitate was collected and further dissolved in 5 mL of DMF, producing the **Ag29-Na3** nanocluster. The yield was 95% based on the Ag element (calculated from the **Ag29-Na1**). This DMF solution of **Ag29-Na3** was directly used for the crystallization and characterization.

### Crystallization of **Ag29-Na1** and **Ag29-Na3**

Single crystals of **Ag29-Na1** or **Ag29-Na3** were cultivated at room temperature by vapor-diffusing ethyl ether into the NMP solution of **Ag29-Na1** or the DMF solution of **Ag29-Na3**. After 2 weeks, red crystals were collected, and the structure of **Ag29-Na1** or **Ag29-Na3** was determined.

### Micellization of **Ag29-Na1** and **Ag29-Na3**

Specifically, 3 mg of **Ag29-Na1** or **Ag29-Na3** was dissolved in 3 mL H_2_O. In this process, **Ag29-Na1** and **Ag29-Na3** cluster-based micelles were formed in their aqueous solutions. For measuring the sizes of **Ag29-Na1** or **Ag29-Na3** micelles in different stages (Fig. 3), 100*n μL of the aforementioned aqueous solution of Ag_29_-based micelles was injected into (3–0.1*n) mL H_2_O each time (*n* is the number of the stage), and the obtained H_2_O solution was directly used for the DLS analysis.

**Fig. 1:**
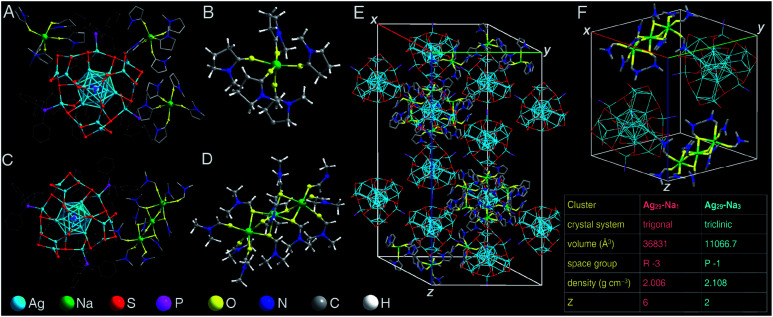
Atomically precise structures of **Ag29-Na1** and **Ag29-Na3**. (A) Crystal structure of **Ag29-Na1**. (B) Structure of the solvent-conjoined cation [Na_1_(NMP)_5_]^+^. (C) Crystal structure of **Ag29-Na3**. (B) Structure of the solvent-conjoined cation [Na_3_(DMF)_12_]^3+^. (E) Crystal lattice of **Ag29-Na1**. (F) Crystal lattice of **Ag29-Na3**. Inset: comparison of crystal data between **Ag29-Na1** and **Ag29-Na3**. Color codes: light blue sphere, Ag; green sphere, Na; red sphere, S; purple sphere, P; yellow sphere, O; blue sphere, N; grey sphere, C; white sphere, H.

**Fig. 2:**
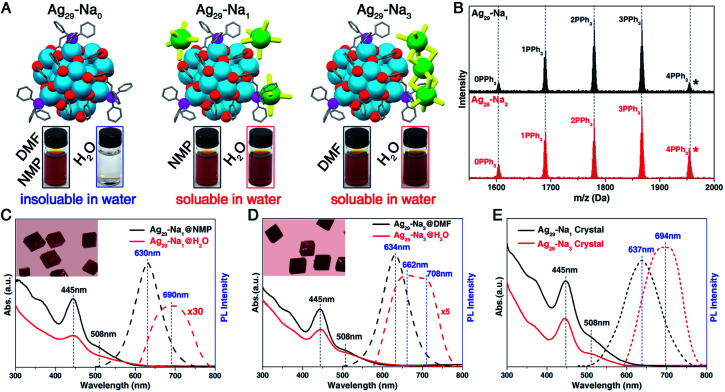
Characterization of **Ag29-Na1** and **Ag29-Na3** nanoclusters. (A) Structures of **Ag29-Na0**, **Ag29-Na1**, and **Ag29-Na3** nanoclusters, and their dissolvability in DMF, NMP or H_2_O solutions. (B) ESI-MS results of **Ag29-Na1** and **Ag29-Na3** nanoclusters. The five peaks in both mass spectra correspond to the Ag_29_(SSR)_12_(PPh_3_)_*n*_ compounds where *n* is 0–4. The peak labelled with “*” matches the integral composition of Ag_29_(SSR)_12_(PPh_3_)_4_. (C) Optical absorptions and emissions of **Ag29-Na1** in NMP or H_2_O solutions. Insets: digital photo of the crystals of **Ag29-Na1**. (D) Optical absorptions and emissions of **Ag29-Na3** in DMF or H_2_O solutions. Insets: digital photo of the crystals of **Ag29-Na3**. (E) Optical absorptions and emissions of **Ag29-Na1** and **Ag29-Na3** nanoclusters in the crystalline state.

**Fig. 3:**
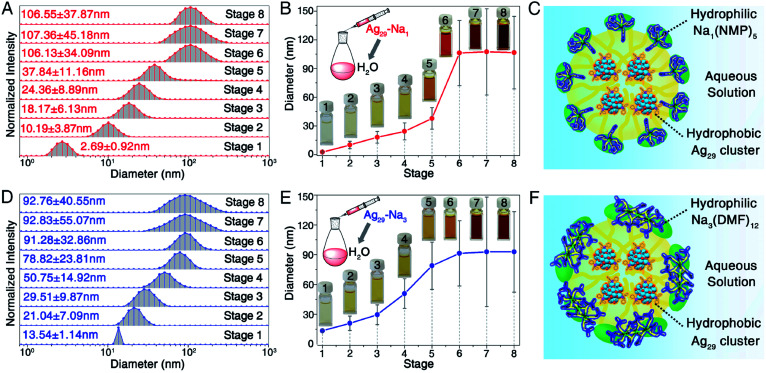
Micellization of **Ag29-Na1** and **Ag29-Na3** nanoclusters. (A and B) Measured sizes of **Ag29-Na1** micelles in different stages. Insets in (B): digital photos of the aqueous solution of **Ag29-Na1** micelles. (C) Scheme illustration of the formation of the **Ag29-Na1** micelle. (D and E) Measured sizes of **Ag29-Na3** micelles in different stages. Insets in (E): digital photos of the aqueous solution of **Ag29-Na3** micelles. (F) Schematic illustration of the formation of the **Ag29-Na3** micelle.

### The general nanocluster micellization method

Specifically, 30 mg of each negative-charged nanocluster was mixed with 1 mg of CH_3_COONa and 30 μL DMF, and the obtained mixture was dissolved in 1 mL H_2_O. The precipitate was then removed to produce the cluster-based micelle in H_2_O. Notably, DLS measurements in Fig. 6 were performed in saturated aqueous solutions of these nanoclusters. The syntheses of negative-charged nanoclusters (including [Au_25_(SC_2_H_4_Ph)_18_]^−^, [Ag_25_(SPhMe_2_)_18_]^−^, [Pt_1_Ag_24_(SPhMe_2_)_18_]^2−^, [Ag_44_(SPhF_2_)_30_]^4−^, [Au_12_Ag_32_(SPhF_2_)_30_]^4−^, and [Ag_28_Cu_12_(SPhCl_2_)_24_]^4−^) were based on the reported methods.^54–58^

**Fig. 4:**
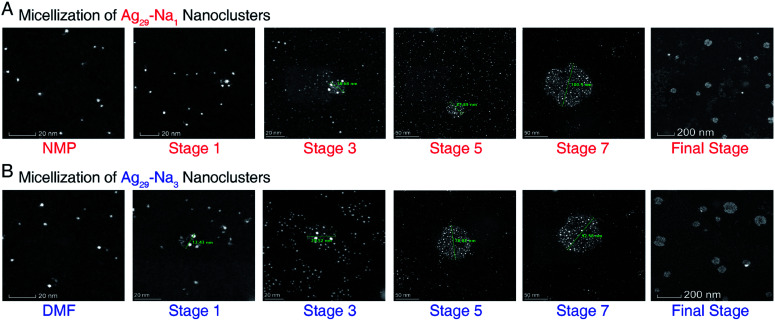
The aberration-corrected HAADF-STEM images of **Ag29-Na1** and **Ag29-Na3** micelles. (A) Micellization of **Ag29-Na1** nanoclusters, corresponding to the different states in Fig. 3A and B. (B) Micellization of **Ag29-Na3** nanoclusters, corresponding to the different states in Fig. 3D and E. Only images of selected stages are shown here, and the whole process images are depicted in ESI Fig. S5 and S6.† Scale bar = 20 nm for NMP/DMF, stage 1, and stage 3; scale bar = 50 nm for stage 5 and stage 7; scale bar = 200 nm for the final stage.

**Fig. 5:**
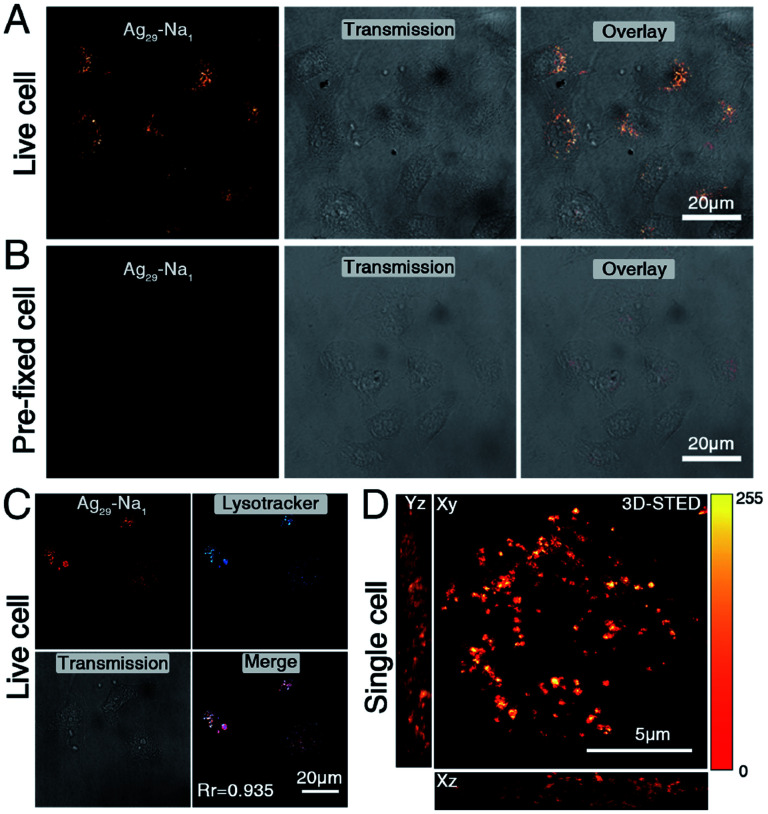
Cell-labelling of the **Ag29-Na1** nanocluster. (A and B) 5 mg mL^−1^**Ag29-Na1** incubated with (A) live and (B) pre-fixed HepG2 cells for 2 hours and imaged under a confocal microscope. Excitation wavelength = 470 nm, emission wavelength = 550–600 nm. Scale bar = 20 μm. (C) 5 mg mL^−1^**Ag29-Na1** incubated with live HepG2 cells and co-stained with Lysotracker Deep Red. The Pearson correlation coefficient (*R*_r_) is 0.935. Scale bar = 20 μm. (D) Three-dimensional (3D) micrograph of single cells incubated with **Ag29-Na1** and imaged under a STED nanoscope. Scale bar = 5 μm.

**Fig. 6:**
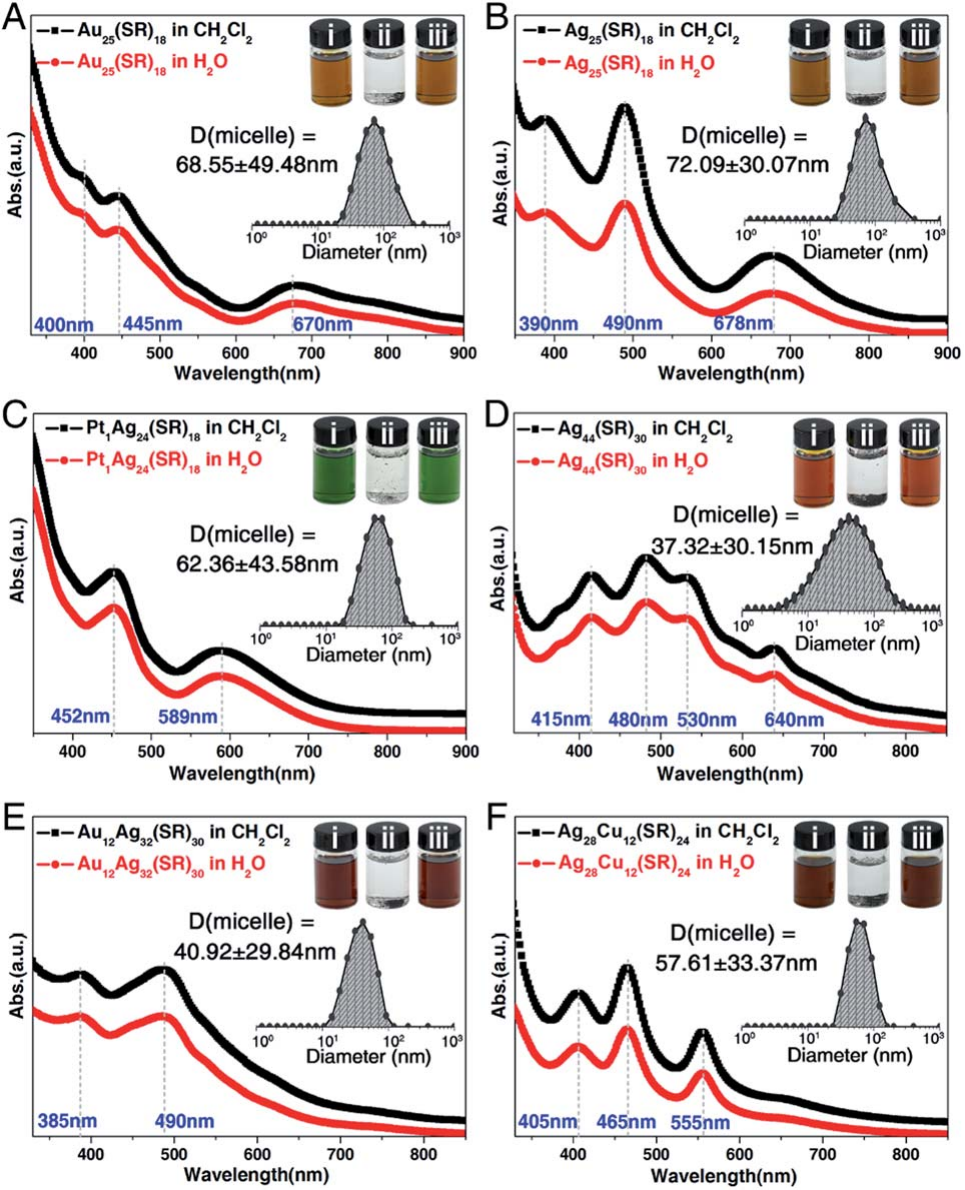
General applicability of the nanocluster micellization method. Optical absorptions of (A) [Au_25_(SC_2_H_4_Ph)_18_]^−^, (B) [Ag_25_(SPhMe_2_)_18_]^−^, (C) [Pt_1_Ag_24_(SPhMe_2_)_18_]^2−^, (D) [Ag_44_(SPhF_2_)_30_]^4−^, (E) [Au_12_Ag_32_(SPhF_2_)_30_]^4−^, and (F) [Ag_28_Cu_12_(SPhCl_2_)_24_]^4−^ nanoclusters in CH_2_Cl_2_ (black lines) and of the corresponding (cluster)^−^(Na–DMF)^+^ micelles in the aqueous phase (red lines). Insets: measured sizes of cluster-based micelles in their size-steady state; digital photos of the nanoclusters: (i) nanoclusters are soluble in CH_2_Cl_2_; (ii) nanoclusters are insoluble in H_2_O; (iii) nanoclusters are soluble in H_2_O in the presence of DMF-conjoined Na^+^ cations.

### The solubility of different nanoclusters in aqueous solution

The nanocluster@solvent-conjoined cation compound was dissolved in H_2_O, and the solubility of each nanocluster in the aqueous solution was determined. Specifically, the solubility of **Ag29-Na1** in H_2_O was 6.76 mg mL^−1^; the solubility of **Ag29-Na3** in H_2_O was 7.88 mg mL^−1^; the solubility of Au_25_(SC_2_H_4_Ph)_18_@Na–DMF in H_2_O was 5.35 mg mL^−1^; the solubility of Ag_25_(SPhMe_2_)_18_@Na–DMF in H_2_O was 5.78 mg mL^−1^; the solubility of Pt_1_Ag_24_(SPhMe_2_)_18_@Na–DMF in H_2_O was 13.42 mg mL^−1^; the solubility of Ag_44_(SPhF_2_)_30_@Na–DMF in H_2_O was 27.12 mg mL^−1^; the solubility of Au_12_Ag_32_(SPhF_2_)_30_@Na–DMF in H_2_O was 28.34 mg mL^−1^; the solubility of Ag_28_Cu_12_(SPhCl_2_)_24_@Na–DMF in H_2_O was 25.32 mg mL^−1^.

### Characterization

All UV-vis absorption spectra of nanoclusters were recorded using an Agilent 8453 diode array spectrometer, whose background correction was made using a pure solution blank.

PL spectra were measured on a FL-4500 spectrofluorometer with the same optical density of 0.1.

Electrospray ionization mass spectrometry (ESI-MS) measurements were performed using a MicrOTOF-QIII high resolution mass spectrometer. The sample was directly infused into the chamber at 5 μL min^−1^. For preparing the ESI samples, nanoclusters were dissolved in NMP/DMF (0.1 mg mL^−1^) and diluted (v/v = 1 : 2) with methanol.

^23^Na nuclear magnetic resonance (NMR) spectra were acquired using a Bruker 600 Avance III spectrometer equipped with a Bruker BBO multinuclear probe (BrukerBioSpin, Rheinstetten, Germany).

Dynamic light scattering (DLS) was performed with a Malvern Zetasizer Nano ZS instrument. For preparing the DLS samples, the nanocluster@solvent-conjoined cation compounds were dissolved in H_2_O to produce the cluster-based micelles. The DLS result of each nanocluster was repeated 40 times to remove the error.

The Ag_29_-based micelles were imaged with an aberration-corrected HAADF-STEM (high angle annular dark field scanning transmission electron microscope) after the solvent that contained Ag_29_-based micelles was drop-cast onto ultrathin carbon film TEM grids. The microscope employed was a FEI Themis Z. The electron beam energy was 200 kV. The collection angle of the HAADF detector was adjusted to collect signals scattered between 52 (inner angle) and 200 (outer angle) mrad (camera length of 146 mm). The HAADF-STEM image was obtained with Thermo Scientific Velox software using 1024 × 1024 pixels and the dwell time is set to 10 us.

### X-ray crystallography

For **Ag29-Na1**, the data collection for single crystal X-ray diffraction was carried out on a Stoe Stadivari diffractometer under nitrogen flow, using graphite-monochromatized Cu Kα radiation (*λ* = 1.54186 Å). For **Ag29-Na3**, the data collection for the single-crystal X-ray diffraction was carried out on a Bruker Smart APEX II CCD diffractometer under nitrogen flow, using graphite-monochromatized Mo K*α* radiation (*λ* = 0.71073 Å). Data reductions and absorption corrections were performed using the SAINT and SADABS programs, respectively. The electron density was squeezed by Platon. The structure was solved by direct methods and refined with full-matrix least squares on *F*^2^ using the SHELXTL software package. All non-hydrogen atoms were refined anisotropically, and all the hydrogen atoms were set in geometrically calculated positions and refined isotropically using a riding model.

### Cell culture

HepG2 (liver hepatocellular carcinoma) cells were purchased from Shanghai Bioleaf BioBiotech. Co. Ltd. Specifically, the cells were incubated in Dulbecco's Modified Eagle's medium (DMEM) containing 10% FBS and 1% antibiotics (penicillin and streptomycin), and the cells were further maintained at 37 °C in an atmosphere of 5% CO_2_ and 95% air.

### Confocal microscopic imaging

Confocal microscopy imaging was performed with a Leica TCS SP8 confocal microscope with an adjustable white laser (470–700 nm) and 63X/100X oil-immersion objective lens. The incubated cells were excited at 470 nm for **Ag29-Na1**, and 633 nm for Lysotracker Red. The emission signals were collected at 550 nm for **Ag29-Na1**, and 650–700 nm for Lysotracker Deep Red.

### STED super-resolution image

Stimulated emission depletion nanoscopy (STED) experiments were performed under a Leica DMi8 confocal microscope equipped with a Leica TCS SP8 STED-ONE unit. The compound was excited under a STED laser (donut laser: 590 nm), and the emission signals were collected using HyD reflected light detectors (RLDs) with 2048 × 2048 pixel and ×100 scanning speed. The STED micrographs were further processed “deconvolution wizard” function using the Huygens Professional software (version: 16.05) under an authorized license. The area radii were estimated under 0.02 micros with exclusion of 100 absolute back-ground values. The following measurement settings were used: maximum iterations: 40 times; signal-to-noise ratio: 20; quality threshold: 0.05; iteration mode: optimized; brick layout: auto.

## 3 Results and discussion

The preparations of both **Ag29-Na1** and **Ag29-Na3** nanoclusters are shown in the Experimental methods section, and the crystallization of **Ag29-Na1** or **Ag29-Na3** nanoclusters was conducted by vapor-diffusing ethyl ether into the NMP solution of **Ag29-Na1** or the DMF solution of **Ag29-Na3**. Structurally, the overall configuration of the Ag_29_(SSR)_12_(PPh_3_)_4_ framework in both **Ag29-Na1** and **Ag29-Na3** was much like that of the previously reported one;^52,53^ however, there were still subtle differences among these Ag_29_ frameworks in terms of the corresponding bond lengths (Table S1†). Specifically, except for the bonds between Ag(core shell) and S(motif), all bonds including Ag(core)–Ag(core shell), Ag(core shell)–Ag(core shell), Ag(core shell)–Ag(motif), Ag(motif)–S(motif), and Ag(motif)–P(motif) of **Ag29-Na1** and **Ag29-Na3** nanoclusters were much longer than those in the bare Ag_29_(SSR)_12_(PPh_3_)_4_ nanocluster (**Ag29-Na0** hereafter). Accordingly, the overall Ag_29_ core structures of both **Ag29-Na1** and **Ag29-Na3** were more expanding than that in **Ag29-Na0**.

For the counterions of these [Ag_29_(SSR)_12_(PPh_3_)_4_]^3−^ nanoclusters, the **Ag29-Na1** nanocluster comprised three NMP-conjoined [Na_1_(NMP)_5_]^+^ cations per Ag_29_ compound (Fig. 1A and B), whereas each Ag_29_ compound matched only one DMF-conjoined [Na_3_(DMF)_12_]^3+^ cation in the structure of **Ag29-Na3** (Fig. 1C and D). It should be noted that no Na^+^ cation (or other cations) has been observed in the crystal lattice of **Ag29-Na0** in both crystals of Ag_29_(SSR)_12_(PPh_3_)_4_ reported by the Bakr and the Pradeep groups, which might result from the high disorder of these Na^+^ counterions.^52,53^ Indeed, induced by the fixation of the crown ether, Chakraborty *et al.* captured these Na^+^ cations in a form of Na^+^@dibenzo-18-crown-6.^59^ In this work, for both **Ag29-Na1** and **Ag29-Na3** nanoclusters, the Na^+^ counterions were fixed by oxygen-carrying solvents such as NMP and DMF to generate the solvent-conjoined cations.

Specifically, for the [Na_1_(NMP)_5_]^+^ of **Ag29-Na1**, each Na^+^ cation was surrounded by five NMP solvent molecules through Na–O interactions (Fig. 1B), whereas for the [Na_3_(DMF)_12_]^3+^ of **Ag29-Na3**, the three Na^+^ cations were tied with a linear pattern by twelve DMF solvent molecules (Fig. 1D).

The crystal lattice of **Ag29-Na1** contained six [Ag_29_(SSR)_12_(PPh_3_)_4_]^3−^ anions and 18 [Na_1_(NMP)_5_]^+^ cations (Fig. 1E); by comparison, two [Ag_29_(SSR)_12_(PPh_3_)_4_]^3−^ anions and two [Na_3_(DMF)_12_]^3+^ cations were observed in the unit cell of **Ag29-Na3** (Fig. 1F). In this context, in terms of the molecular charge, each “−3”-charged Ag_29_ matched three “+1”-charged [Na_1_(NMP)_5_]^+^ in **Ag29-Na1**, or one “+3”-charged [Na_3_(DMF)_12_]^3+^ in **Ag29-Na3** to realize the charge balance.

Although the **Ag29-Na0** nanocluster displayed good solubility in NMP or DMF, it was absolutely hydrophobic. However, owing to the presence of the solvent-conjoined cations such as Na_1_(NMP)_5_ and Na_3_(DMF)_12_, **Ag29-Na1** and **Ag29-Na3** nanoclusters were perfectly soluble in both organic reagents (NMP and DMF) and the aqueous solution (Fig. 2A). Previous research has investigated the phase transfer of hydrophilic nanoclusters from aqueous to organic phases induced by the addition of counterions (*e.g.*, phase transfer of the Au_22_ nanocluster in the presence of tetraoctylammonium cations);^60–62^ however, the study of the reverse process (*i.e.*, phase transfer of hydrophobic nanoclusters from organic to aqueous phases) is rather limited in the nanocluster research field. In this work, based on the Ag_29_(SSR)_12_(PPh_3_)_4_ cluster template, the transfer of hydrophobic nanoclusters from the organic phase to water has been accomplished.

ESI-MS results of both **Ag29-Na1** and **Ag29-Na3** nanoclusters exhibited five peaks corresponding to the Ag_29_(SSR)_12_(PPh_3_)_*n*_ compounds where *n* was 0–4 (Fig. 2B and S1‡), demonstrating the dissociation–aggregation pattern of PPh_3_ ligands on the Ag_29_ nanocluster surface.^63^ However, the [Na_1_(NMP)_5_]^+^ and [Na_3_(DMF)_12_]^3+^ were undetectable in the ESI-MS, which was proposed to result from the weak interactions between Na^+^ cations and NMP/DMP molecules causing such solvent-conjoined cations to decompose in mass spectroscopy. ^23^Na NMR was performed to verify the presence of Na^+^ or Na^+^-conjoined cations in these nanoclusters. As shown in ESI Fig. S2A,† the ^23^Na NMP signals of CH_3_COONa were 0.55 and 1.36 ppm in DMF-D7 and NMP-D9, respectively, whereas the signals of **Ag29-Na0** (in DMF-D7), **Ag29-Na1** (in NMP-D9) and **Ag29-Na3** (in DMF-D7) were −3.08, −0.88, and −1.06 ppm, respectively. Such differences also suggested the distinct existing form of each Na^+^-based cation in the corresponding nanocluster. Besides, the ^23^Na NMP signal of CH_3_COONa in D_2_O is located at −0.12 ppm, which was remarkably different from those of **Ag29-Na1** or **Ag29-Na3** in D_2_O (−3.75 or −3.36 ppm, respectively; Fig. S2B†). In this context, throughout the micellization of Ag_29_ clusters, Na^+^/solvent counterions would not dissociate from the nanoclusters. However, it still remained unknown whether the structures of [Na_1_(NMP)_5_]^+^ and [Na_3_(DMF)_12_]^3+^ cations retained in the cluster-based micelles because these micelles were hard to analyze at the atomic level.

The optical absorptions and emissions of the Ag_29_ nanoclusters in different solutions were compared. As depicted in Fig. 2C, the UV-vis spectrum of the NMP solution of **Ag29-Na1** showed an intense absorption at 445 nm and three shoulder bands at 320, 365, and 508 nm, whereas all of these peaks were attenuated when the nanocluster was dissolved in aqueous solution; such a phenomenon was also observed for the **Ag29-Na3** nanocluster (Fig. 2D). Despite this attenuation, the optical absorptions of both **Ag29-Na1** and **Ag29-Na3** in aqueous solution were actually quite similar to those in NMP or DMF. However, remarkable differences existed in terms of the emission wavelength and the photoluminescence (PL) intensity (Fig. 2C and D). Specifically, the **Ag29-Na1**@NMP emitted at 630 nm, whereas the emission peak of **Ag29-Na1**@H_2_O is located at 690 nm along with the broadening of the emission wavelength. Besides, the emission of **Ag29-Na3**@DMF was centered at 634 nm, and the broadened emission of **Ag29-Na3**@H_2_O displayed two peaks at 662 and 708 nm. Of note, compared with the **Ag29-Na1** in NMP or **Ag29-Na3** in DMF, significant attenuation on PL intensity was monitored when these Ag_29_ nanoclusters were dissolved in H_2_O.

Although all types of Ag_29_ nanoclusters (**Ag29-Na0**, **Ag29-Na1**, and **Ag29-Na3**) presented cubic-like crystals at the macro-level (Fig. 2C,D, insets),^52,53^ the emissions of them were entirely different. It has been demonstrated that the crystal of **Ag29-Na0** emitted at 670 or 700 nm with different crystalline patterns.^53^ However, the emissions of **Ag29-Na1** and **Ag29-Na3** luminesced at 637 and 694 nm, respectively (Fig. 2E; and see Fig. S3† for the emission of **Ag29-Na0**). Besides, the PL intensity of the **Ag29-Na3** crystal was slightly stronger than that of the **Ag29-Na1** crystal. Such differences in emission wavelength and PL intensity of these Ag_29_ crystals resulted from their different crystal lattices (or different inter-cluster interactions).

It has been demonstrated that the hydrophobic Ag_29_(SSR)_12_(PPh_3_)_4_ nanoclusters can be transferred into water in the presence of solvent-conjoined Na^+^ cations; however, the existence of these Ag_29_ nanoclusters in aqueous solution remains mysterious. Herein, the DLS (dynamic light scattering) and aberration-corrected HAADF-STEM techniques have been performed for monitoring their real-time existence.

With the help of DLS, the sizes of these Ag_29_ nanoclusters (**Ag29-Na1** and **Ag29-Na3**) in aqueous solution were monitored (Fig. 3). The concentration of **Ag29-Na1** (or **Ag29-Na3**) in H_2_O of each stage was controlled as (0.1*n/3) mg mL^−1^ (*n* is the stage number in Fig. 3, and see the corresponding optical absorptions in Fig. S4†). For **Ag29-Na1**, the measured sizes of these clusters were quite small (∼2.69 nm) when their concentration was low (0.03 mg mL^−1^). When the concentration of nanoclusters increased, a remarkable size growth was observed. Finally, the **Ag29-Na1** cluster size soared to a plateau of ∼106 nm (Fig. 3A and B). A similar variation tendency has been observed for the **Ag29-Na3** nanocluster, whose sizes in the aqueous solutions grew from ∼13.54 nm to ∼92.76 nm (Fig. 3D and E). Considering that it is not possible for the hydrophobic Ag_29_(SSR)_12_(PPh_3_)_4_ clusters to form the contact surface with H_2_O molecules, we proposed that the phase-transfer of Ag_29_ into water resulted from the micellization of such nanoclusters. The critical micelle concentration (CMC) of **Ag29-Na1** and **Ag29-Na3** micelles should both occur at stage 6 (Fig. 3), and thus the CMC values of both Ag_29_ micelles were determined as 0.2 mg mL^−1^.

As depicted in Fig. 3C and F, the Ag_29_ cluster-based micelle was composed of a hydrophobic Ag_29_ interior and a hydrophilic Na–NMP (or Na–DMF) surface. That is, owing to the water-soluble Na–NMP (or Na–DMF) surface, the cluster-based micelle displayed good dissolvability and stability in aqueous solution.

The aberration-corrected HAADF-STEM measurements were further performed to verify the generation of the cluster-based micelles. Fig. 4, S5, and S6† show the selected images of these Ag_29_-carrying micelles. As depicted in Fig. 4A and S5,†**Ag29-Na1** cluster entities were discrete in NMP, and were gradually assembled in aqueous solution. With the increased concentration of the dissolved **Ag29-Na1** in H_2_O, the sizes of micelles increased gradually. Finally, the sizes of the **Ag29-Na1** micelles were stabilized at about 100 nm. Similar size variations have also been observed for the **Ag29-Na3** micellization process, where the final-stage sizes were also determined as about 100 nm (Fig. 4b and S6†). Such a size growth trend and the final-stage size excellently matched with those derived from the DLS measurement (Fig. 3), further confirming the cluster micellization process. Based on the DLS and STEM results, the aggregation numbers in cluster-based micelles were proposed (Fig. S7†)—111 360 of **Ag29-Na1** in each micelle with a 106 nm diameter and 72 810 of **Ag29-Na3** in each micelle with a 92 nm diameter.

Of note, although the structure of the Ag_29_(SSR)_12_(PPh_3_)_4_ molecule is retained during cluster micellization, the structures of [Na_1_(NMP)_5_]^+^ and [Na_3_(DMF)_12_]^3+^ cations may be altered; however, the water solubility of **Ag29-Na1** and **Ag29-Na3** activated by the presence of these solvent-conjoined cations indeed renders these hydrophobic clusters biocompatible to some extent, which sheds light on the preparation of atomically precise cluster-based, biocompatible nanomaterials.

Although both **Ag29-Na1** and **Ag29-Na3** exhibited excellent solubility in water (6.76 mg mL^−1^ for the **Ag29-Na1** and 7.88 mg mL^−1^ for **Ag29-Na3**), the **Ag29-Na3** micelles were prone to coagulation; by comparison, the **Ag29-Na1** micelles were quite stable in the aqueous phase (Fig. S8†). Due to the excellent stability of **Ag29-Na1** micelles, their performance in cell staining was evaluated. Specifically, 5 mg mL^−1^**Ag29-Na1** was incubated with live HepG2 cells and imaged directly under a laser confocal microscope. As shown in Fig. 5A, after incubation for 2 hours, **Ag29-Na1** enabled effective uptake in the cytosolic region and displayed a punctate signal. In a parallel experiment (Fig. 5B), the same concentration of **Ag29-Na1** incubated with pre-fixed cells displayed neglected uptake. These results demonstrated that **Ag29-Na1** was not a cell permeable probe, but might be internalized with live cells *via* an energy-dependent uptake pathway, such as endocytosis. To precisely determine the intracellular compartment where **Ag29-Na1** is stained, a colocalization experiment was performed. Live cells were incubated with **Ag29-Na1** and labelled with a lysosomal commercial dye, LysoTracker Deep Red. The micrograph in Fig. 5C suggested that the **Ag29-Na1** signal highly overlapped with the LysoTracker signal with a Pearson correlation coefficient (*R*_r_) of 0.935, which further confirmed that the cell entry of **Ag29-Na1** might follow an endocytosis pathway. These observations also demonstrated that the **Ag29-Na1** micelles could stain lysosomes.

Furthermore, the bio-application of **Ag29-Na1** in super-resolution imaging was evaluated. The chosen single cell was incubated with **Ag29-Na1** as described above and imaged under a stimulated emission depletion nanoscope (STED). The three-dimensional (3D) micrographs revealed a whole cell lysosome distribution at an unprecedented resolution at both the *x*-axis and *z*-axis. This strongly demonstrated the ultra-high photon-stability of **Ag29-Na1** and reflected that such cluster micelles could be utilized for super-resolution-based imaging. Of note, due to the existence of the lipophilic phospholipid bilayer in the cell, the structures of [Na_1_(NMP)_5_]^+^ and [Na_3_(DMF)_12_]^3+^ cations were hard to retain in the cell staining process. However, the significant role of these solvent-conjoined cations in corresponding Ag_29_ nanoclusters was the phase-transfer effect that rendered these hydrophobic nanoclusters water-soluble, and thus their bio-applications appear to be promising.

In previous studies concerning cation-containing micelles, the role of Na^+^ cations in micellization has been thoroughly researched (*e.g.*, the micellization of alkyl sulfates or the ionic micelles).^64–68^ However, the cation-induced micellization has not been reported in the nanocluster field. Considering that the solvent-conjoined Na^+^ cations can act as general counterions for negatively charged nanoclusters, we perceive a good opportunity to render such hydrophobic nanoclusters water-soluble. Herein, several negative-charged nanoclusters including [Au_25_(SC_2_H_4_Ph)_18_]^−^, [Ag_25_(SPhMe_2_)_18_]^−^, [Pt_1_Ag_24_(SPhMe_2_)_18_]^2−^, [Ag_44_(SPhF_2_)_30_]^4−^, [Au_12_Ag_32_(SPhF_2_)_30_]^4−^, and [Ag_28_Cu_12_(SPhCl_2_)_24_]^4−^ nanoclusters were used for evaluating the general applicability of the nanocluster micellization strategy.^54–58^ For the preparation, each nanocluster was mixed with CH_3_COONa and minute quantities of DMF, which produced the [cluster]^−^[Na–solvent]^+^ compounds. As shown in the digital photos in Fig. 6, in the absence of [Na–solvent]^+^ cations, these nanoclusters were well soluble in CH_2_Cl_2_ (photo i) but insoluble in H_2_O (photo ii); that is, they were absolutely hydrophobic. By comparison, in the presence of solvent-conjoined Na^+^ cations, all of the obtained compounds showed good dissolvability in aqueous solution (photo iii). Specifically, the water solubility of Au_25_(SC_2_H_4_Ph)_18_@Na–DMF, Ag_25_(SPhMe_2_)_18_@Na–DMF, Pt_1_Ag_24_(SPhMe_2_)_18_@Na–DMF, Ag_44_(SPhF_2_)_30_@Na–DMF, Au_12_Ag_32_(SPhF_2_)_30_@Na–DMF, Ag_28_Cu_12_(SPhCl_2_)_24_@Na–DMF was 5.35, 5.78, 13.42, 27.12, 28.34, and 25.32 mg mL^−1^, respectively. As depicted in Fig. 6, for each nanocluster, the optical absorptions in CH_2_Cl_2_ and in H_2_O were the same, demonstrating the stability of the nanocluster in the aqueous phase. Furthermore, DLS measurements were performed in the saturated aqueous solutions of these nanoclusters, and all of the size-distribution results demonstrated the generation of cluster-based micelles (Fig. 6, insets). Consequently, the micellization of nanoclusters triggered by the addition of solvent-conjoined cations is indeed a generally applicable strategy for rendering hydrophobic nanoclusters water-soluble, at least for the negatively charged nanoclusters.

## 4 Conclusions

In summary, we presented a versatile strategy to render hydrophobic nanoclusters water-soluble—the micellization of nanoclusters; such a dissolvability variation was triggered by the addition of solvent-conjoined Na^+^ cations. Specifically, although several negative-charged nanoclusters (such as Ag_29_(SSR)_12_(PPh_3_)_4_, Au_25_(SR)_18_, Ag_25_(SR)_18_, *etc.*) were absolutely hydrophobic, they showed good dissolvability in aqueous solution in the presence of solvent-conjoined Na^+^ cations. Crystal structures of **Ag29-Na1** and **Ag29-Na3** demonstrated that such Na^+^ cations were capped by oxygen-carrying solvent molecules, and existed as [Na_1_(NMP)_5_]^3+^ or [Na_3_(DMF)_12_]^3+^, acting as both counterions of negatively charged nanoclusters and surface cosolvent of cluster-based micelles in the aqueous phase. A combination of DLS and aberration-corrected HAADF-STEM unambiguously identified the generation of micelles of such nanoclusters. Owing to the excellent water solubility and stability of **Ag29-Na1**, its performance in cell staining has been evaluated—**Ag29-Na1** cluster-based micelles can stain lysosomes in both general imaging and super-resolution-based imaging. Overall, this work hopefully sheds light on the preparation of atomically precise cluster-based, biocompatible nanomaterials.

## Conflicts of interest

There are no conflicts to declare.

## Supplementary Material

SC-011-D0SC01055C-s001

SC-011-D0SC01055C-s002
