# Supplementary material for: Rendering hydrophobic nanoclusters water-soluble and biocompatible
Source: Chem Sci. 2020 Apr 22;11(18):4808–16. doi: 10.1039/d0sc01055c (PMC8159227; doi:10.1039/d0sc01055c)
Supplement: SC-011-D0SC01055C-s001 [file SC-011-D0SC01055C-s001.pdf]

Electronic Supplementary Material (ESI) for Chemical Science.
This journal is © The Royal Society of Chemistry 2020

Supporting Information

Rendering Hydrophobic Nanoclusters Water-soluble and Biocompatible

Xi Kang,[‡,a] Xiao Wei,[‡,a] Pan Xiang,[b] Xiaohe Tian,[b] Zewen Zuo,[c,d] Fengqi Song,[c,d] Shuxin Wang*[,a] and Manzhou Zhu*[,a]

[a]Department of Chemistry and Centre for Atomic Engineering of Advanced Materials, Anhui Province Key Laboratory of Chemistry for Inorganic/Organic Hybrid Functionalized Materials, Key Laboratory of Structure and Functional Regulation of Hybrid Materials of Ministry of Education, Anhui University, Hefei 230601, P. R. China.
[b]School of Life Sciences, Anhui University, Hefei 230601, P. R. China.
[c]National Laboratory of Solid State Microstructures, Collaborative Innovation Center of Advanced Microstructures, School of Physics, Nanjing University, Nanjing 210093, P. R. China.
[d]Atomic Manufacture Institute, Nanjing 211805, P. R. China.

[‡]X.K. and X.W. contributed equally.

*E-mails of corresponding authors: ixing@ahu.edu.cn (S.W.); zmz@ahu.edu.cn (M.Z.).

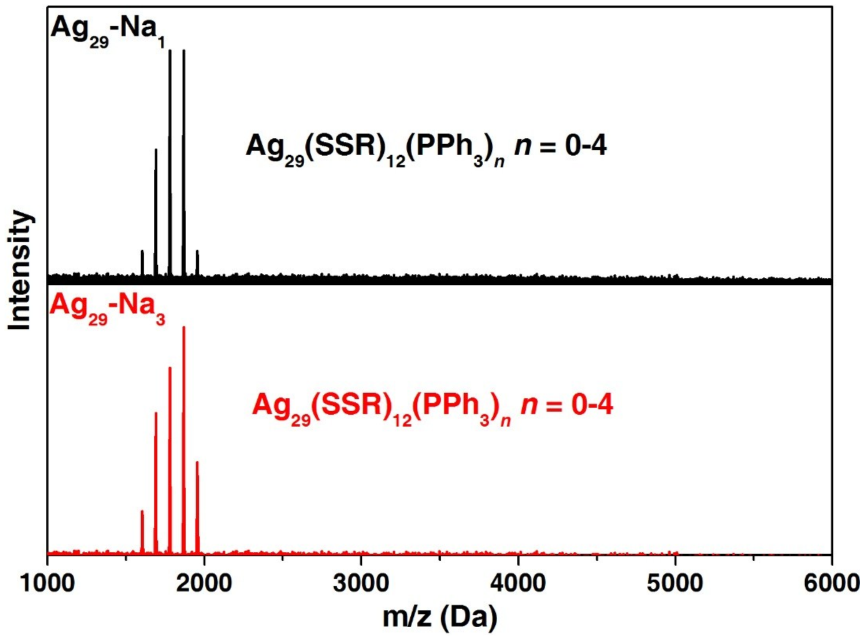

Fig. S1 ESI-MS results of nanoclusters. ESI-MS results of **Ag_{29}-Na_1** and **Ag_{29}-Na_3** nanoclusters, in the range from 1000 to 6000 Da. The five peaks in both mass spectra correspond to the $Ag_{29}(SSR)_{12}(PPh_3)_n$ compounds where n = 0-4.

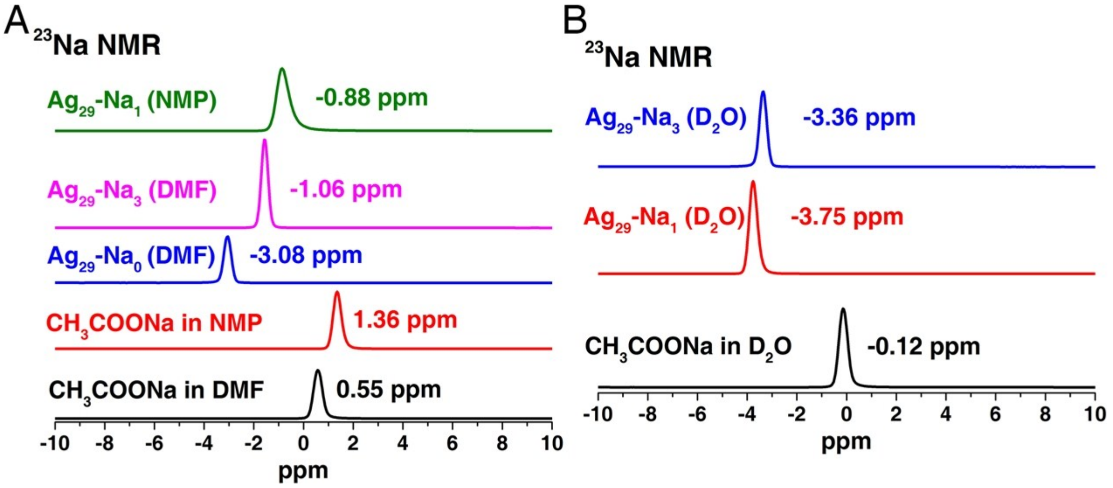

Fig. S2 ^{23}Na NMR spectra of nanoclusters. (A) ^{23}Na NMR spectra of CH_3COONa (black), **Ag_{29}-Na_0** (blue), and **Ag_{29}-Na_3** (magenta) in DMF-D7, and CH_3COONa (red) and **Ag_{29}-Na_1** (green) in NMP-D9. (B) ^{23}Na NMR spectra of CH_3COONa (black), **Ag_{29}-Na_1** (red), and **Ag_{29}-Na_3** (blue) in D_2O.

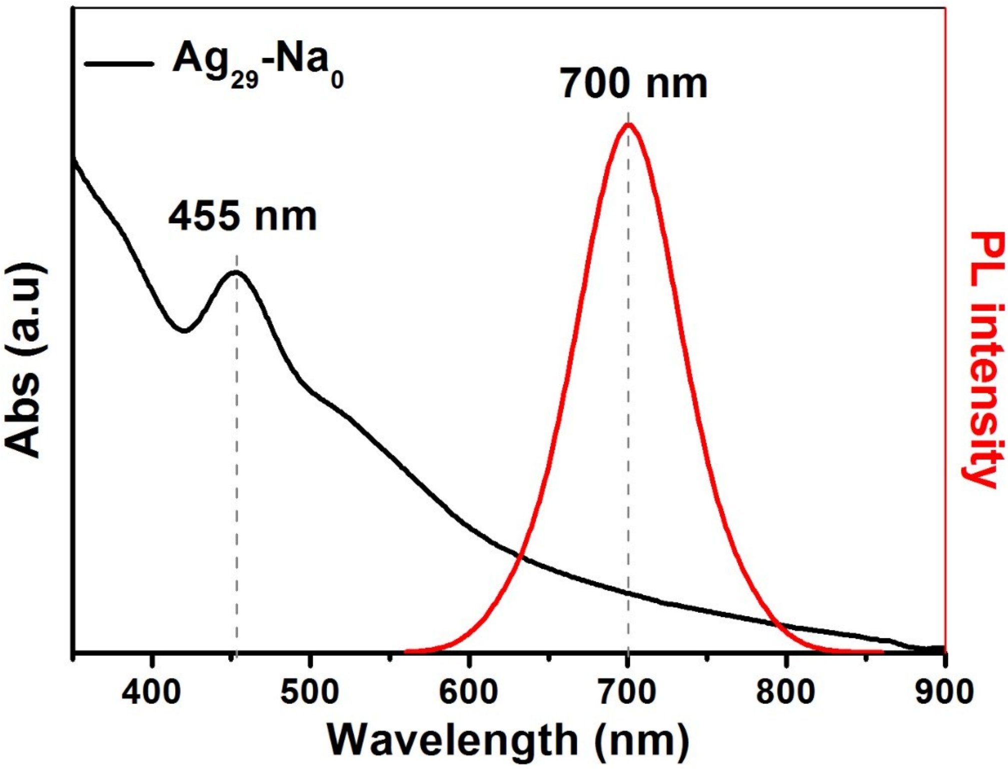

Fig. S3 Optical absorption and emission of **Ag_{29}-Na_0**. Optical absorption and emission of **Ag_{29}-Na_0** nanocluster in the crystalline state (with a drop-cast crystallization mode).

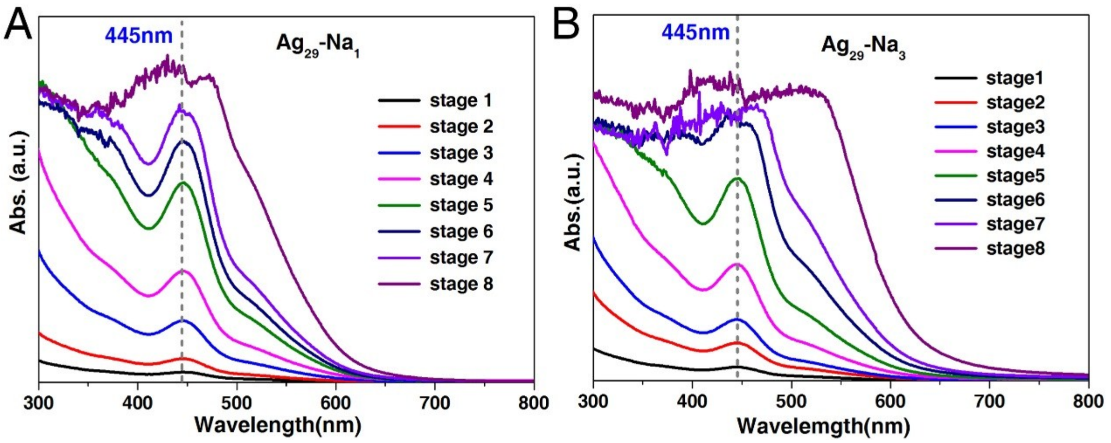

Fig. S4 Nanocluster concentration-dependent optical absorptions. Nanocluster concentration-dependent optical absorptions of (**A**) **Ag_{29}-Na_1** and (**B**) **Ag_{29}-Na_3** nanoclusters in H_2O. These spectra correspond to the stages in Fig. 3.

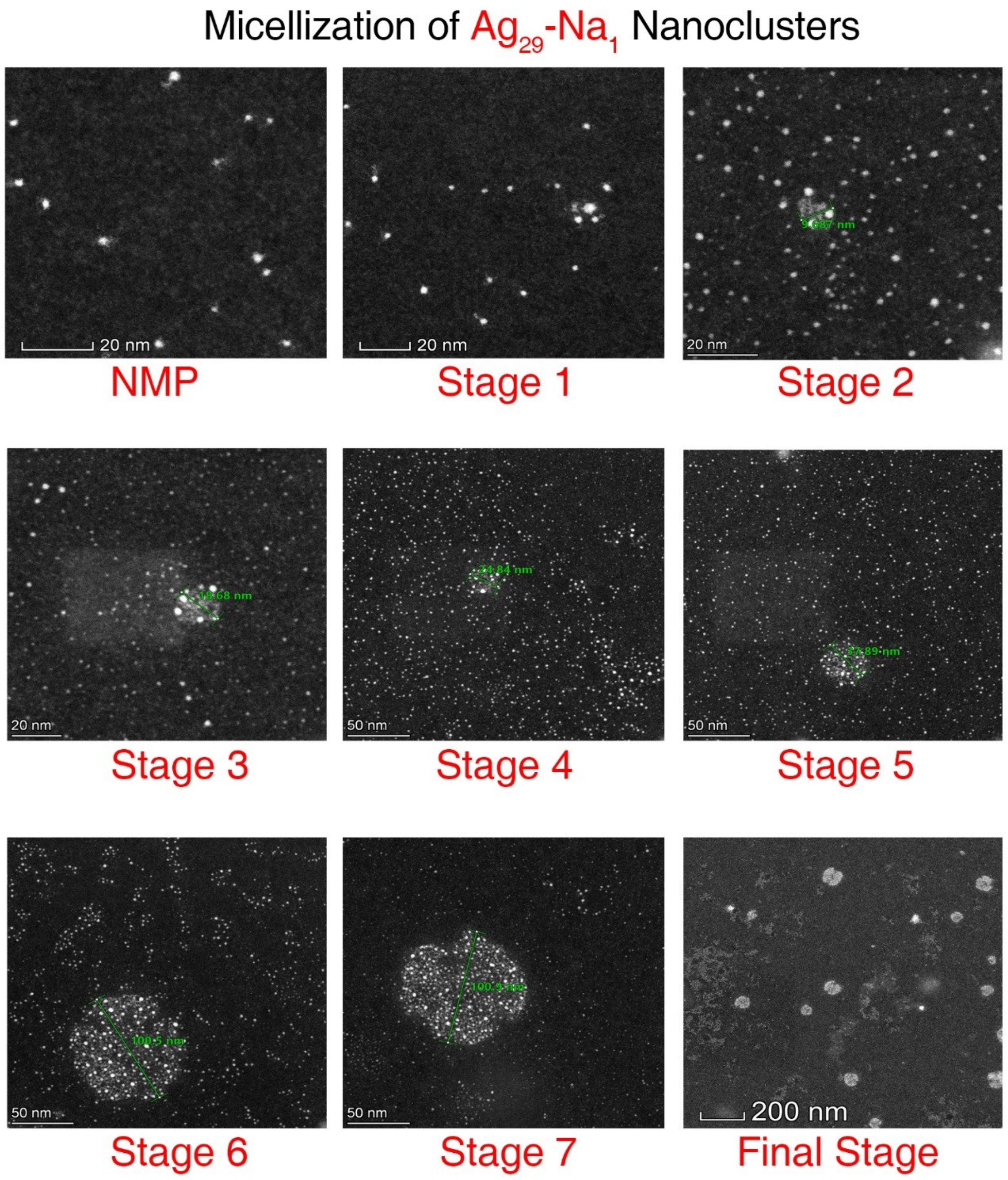

Fig. S5 Aberration-corrected HAADF-STEM images of the micellization of **Ag_{29}-Na_1**. The aberration-corrected HAADF-STEM images of the micellization of **Ag_{29}-Na_1** nanoclusters, corresponding to the different states in Fig. 3A,B. The scale bar of the NMP solution and the stages 1-3 is 20 nm; the scale bar of the stages 4-7 is 50 nm; the scale bar of the final stage is 200 nm.

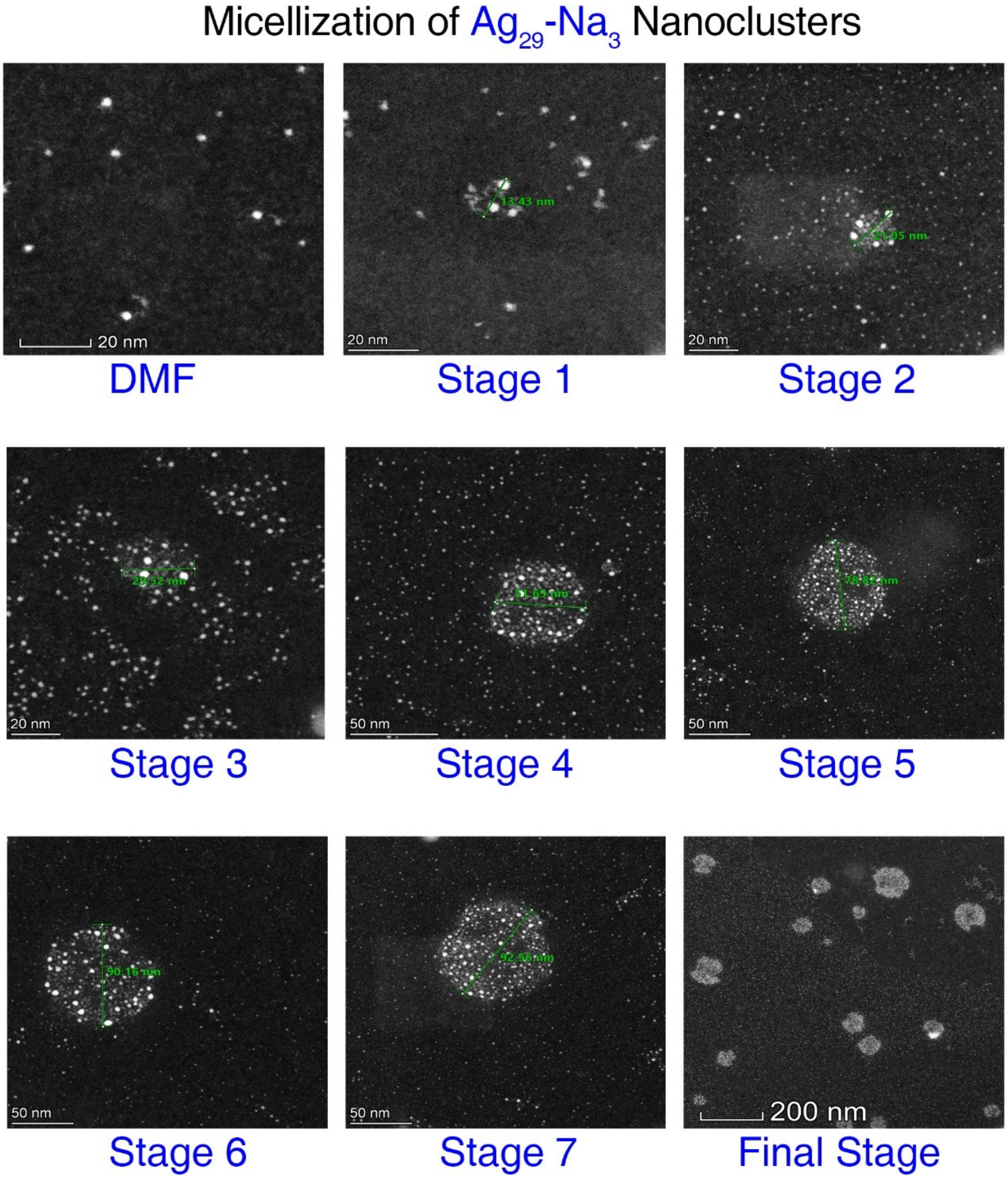

Fig. S6 Aberration-corrected HAADF-STEM images of the micellization of **Ag_{29}-Na_3**. The aberration-corrected HAADF-STEM images of the micellization of **Ag_{29}-Na_3** nanoclusters, corresponding to the different states in Fig. 3D,E. The scale bar of the NMP solution and the stages 1-3 is 20 nm; the scale bar of the stages 4-7 is 50 nm; the scale bar of the final stage is 200 nm.

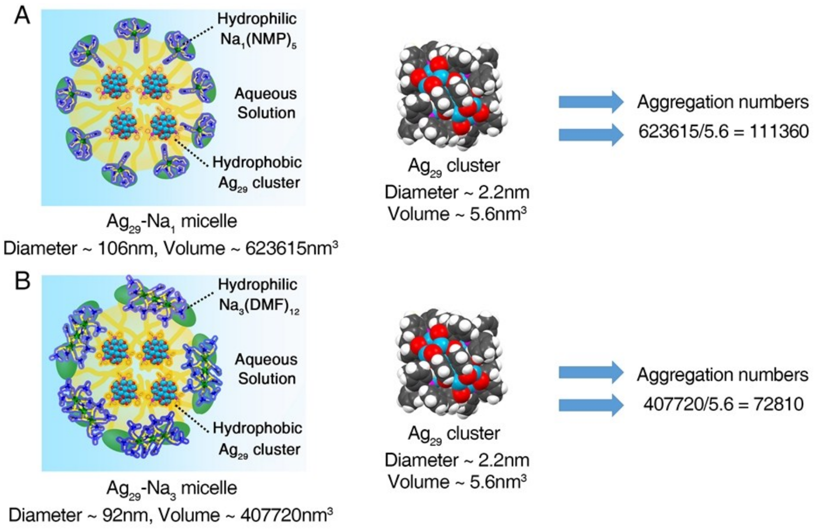

Fig. S7 Proposed aggregation numbers of **Ag_{29}-Na_1** or **Ag_{29}-Na_3** nanoclusters in their corresponding micelles — (A) 111360 of **Ag_{29}-Na_1** in each micelle (106 nm, according to Fig. 3A,B) and (B) 72810 of **Ag_{29}-Na_3** in each micelle (92 nm, according to Fig. 3D,E). In order to simplify the calculation, both the cluster and the micelle are regarded as the standard spheres. *Of note, the actual aggregation numbers should be less than the calculated ones because the cluster molecules cannot be compactly aggregated in the corresponding micelles.*

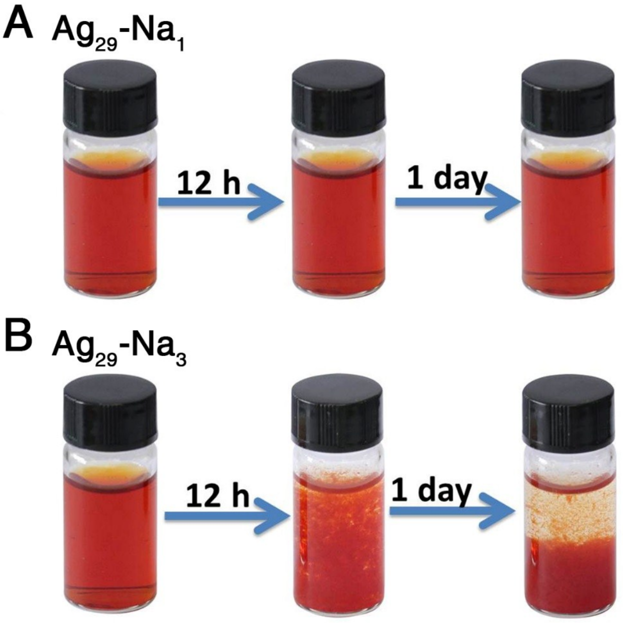

Fig. S8 Stability of **Ag_{29}-Na_1** and **Ag_{29}-Na_3**. The stability of (**A**) **Ag_{29}-Na_1** and (**B**) **Ag_{29}-Na_3** nanoclusters in H_2O. The **Ag_{29}-Na_1** is stable within 1 day; by comparison, the aqueous solution of **Ag_{29}-Na_3** is unstable within 12 hours to produce red flocculent precipitates. In this context, the aqueous solution of **Ag_{29}-Na_1** is more suitable for the biological application.

Table S1. Comparison of bond lengths of nanoclusters.

Cluster		**Ag_{29}-Na_0**	**Ag_{29}-Na_1**	Diff.	**Ag_{29}-Na_3**	Diff
Ag(core)-Ag(core shell)[a]	Range (Å)	2.755-2.772	2.753-2.792		2.750-2.794	
	Avg.(Å)	2.765	2.770	+0.18%	2.772	+0.25%
Ag(core shell)-Ag(core shell)[b]	Range (Å)	2.834-2.975	2.830-2.992		2.821-3.012	
	Avg.(Å)	2.907	2.912	+0.17%	2.915	+0.28%
Ag(core shell)-S(motif)[c]	Range (Å)	2.451-2.474	2.445-2.479		2.448-2.472	
	Avg.(Å)	2.460	2.460	0	2.459	-0.04%
Ag(core shell)-Ag(motif)[d]	Range (Å)	3.077-3.158	3.033-3.260		3.017-3.248	
	Avg.(Å)	3.111	3.116	+0.16%	3.121	+3.21%
Ag(motif)-S(motif)[e]	Range (Å)	2.433-2.612	2.452-2.645		2.444-2.650	
	Avg.(Å)	2.522	2.536	+0.56%	2.532	+0.40%
Ag(motif)-P[f]	Range (Å)	2.484-2.491	2.490-2.549		2.486-2.511	
	Avg.(Å)	2.489	2.505	+0.64%	2.497	+0.32%

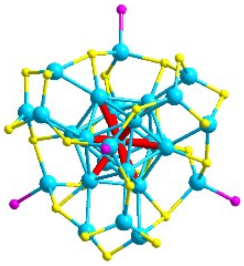

a: bonds between Ag(core) and Ag(core shell), highlighted in red.

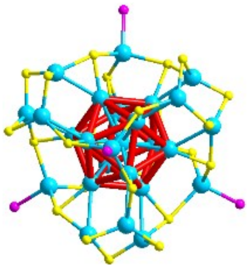

b: bonds between Ag(core shell) and Ag(core shell), highlighted in red.

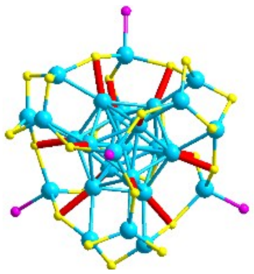

c: bonds between Ag(core shell) and S(motif), highlighted in red.

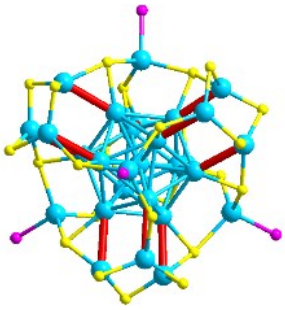

d: bonds between Ag(core shell) and Ag(motif), highlighted in red.

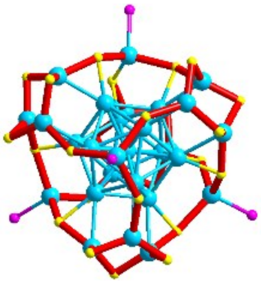

e: bonds between Ag(motif) and S(motif), highlighted in red.

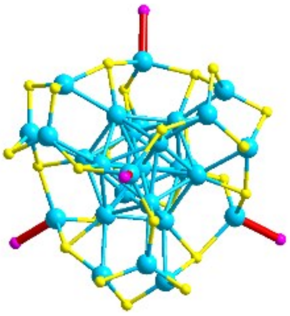

f: bonds between Ag(motif) and P, highlighted in red.

Table S2. Crystal data and structure refinement for the Ag_{29}-Na_1.

Crystal system	trigonal
Space group	*R* -3
a/Å	26.7522(10)
b/Å	26.7522(10)
c/Å	59.424(3)
α/°	90
β/°	90
γ/°	120
Volume/Å^3	36831(3)
Z	6
ρ_{calc}g/cm^3	2.006
μ/mm^{-1}	20.739
F(000)	21732
Radiation	CuKα (λ = 1.54186)
Index ranges	-28 ≤ h ≤ 31, -32 ≤ k ≤ 22, -71 ≤ l ≤ 53
Final R indexes [I>=2σ (I)]	R_1 = 0.0716, wR_2 = 0.1670
Final R indexes [all data]	R_1 = 0.1260, wR_2 = 0.2067

Table S3. Crystal data and structure refinement for the Ag_{29}-Na_3.

Crystal system	triclinic
Space group	P -1
a/Å	22.5850(15)
b/Å	23.5280(17)
c/Å	24.3365(17)
α/°	70.245(3)
β/°	70.380(3)
γ/°	69.824(3)
Volume/Å^3	11066.7(14)
Z	2
ρ_{calc}g/cm^3	2.108
μ/mm^{-1}	2.817
F(000)	6826
Radiation	MoKα (λ = 0.71073)
Index ranges	-28 ≤ h ≤ 28, -28 ≤ k ≤ 30, -31 ≤ l ≤ 31
Final R indexes [I>=2σ (I)]	R_1 = 0.0402, wR_2 = 0.0954
Final R indexes [all data]	R_1 = 0.0482, wR_2 = 0.0988
